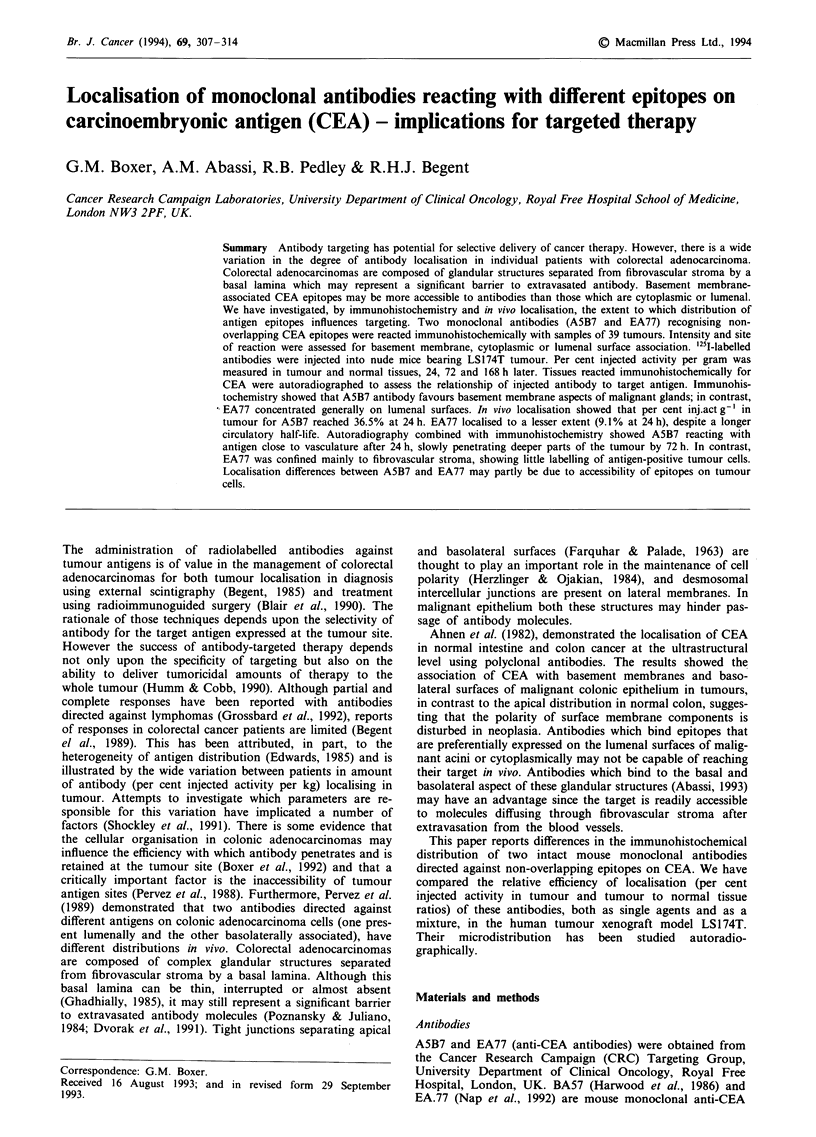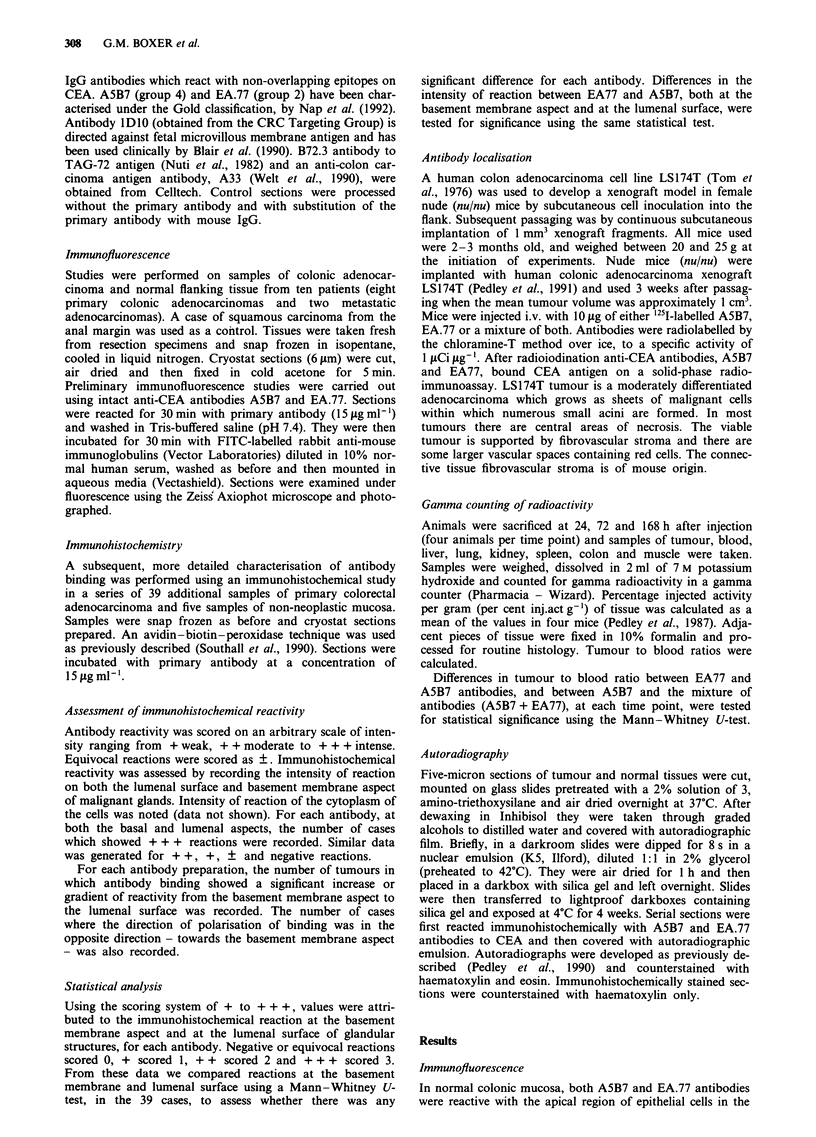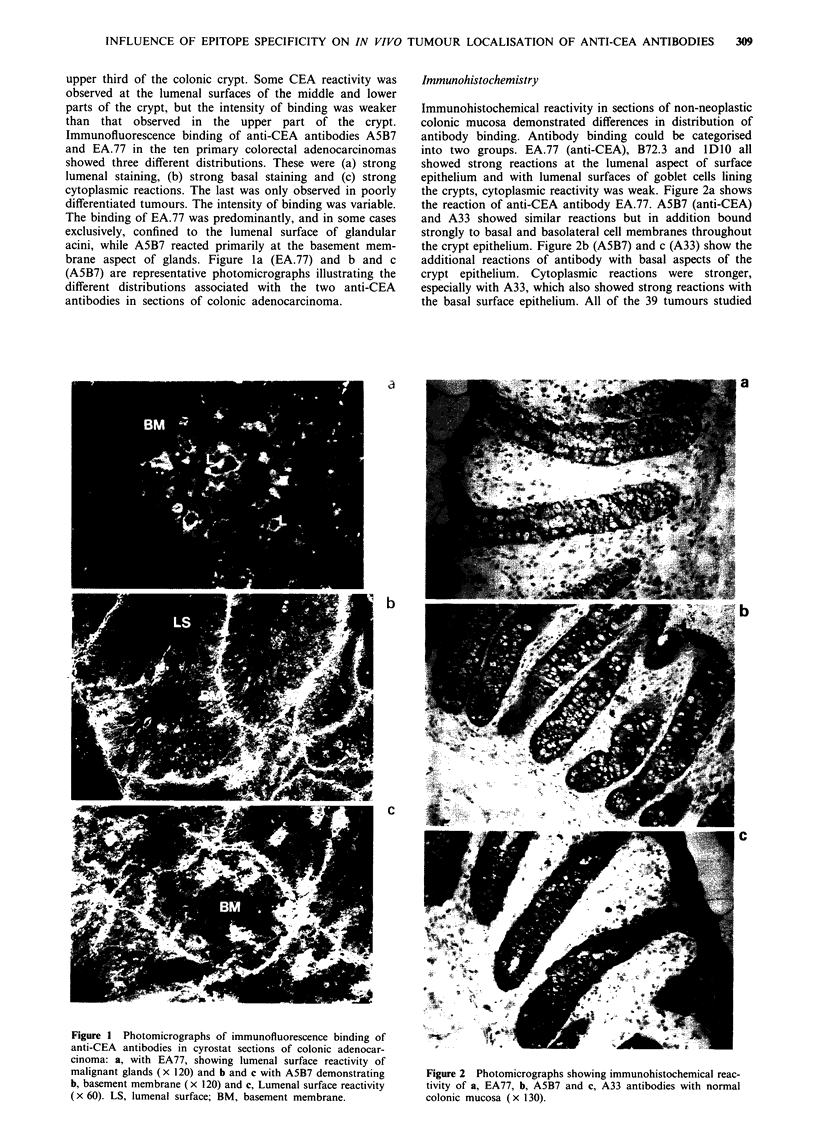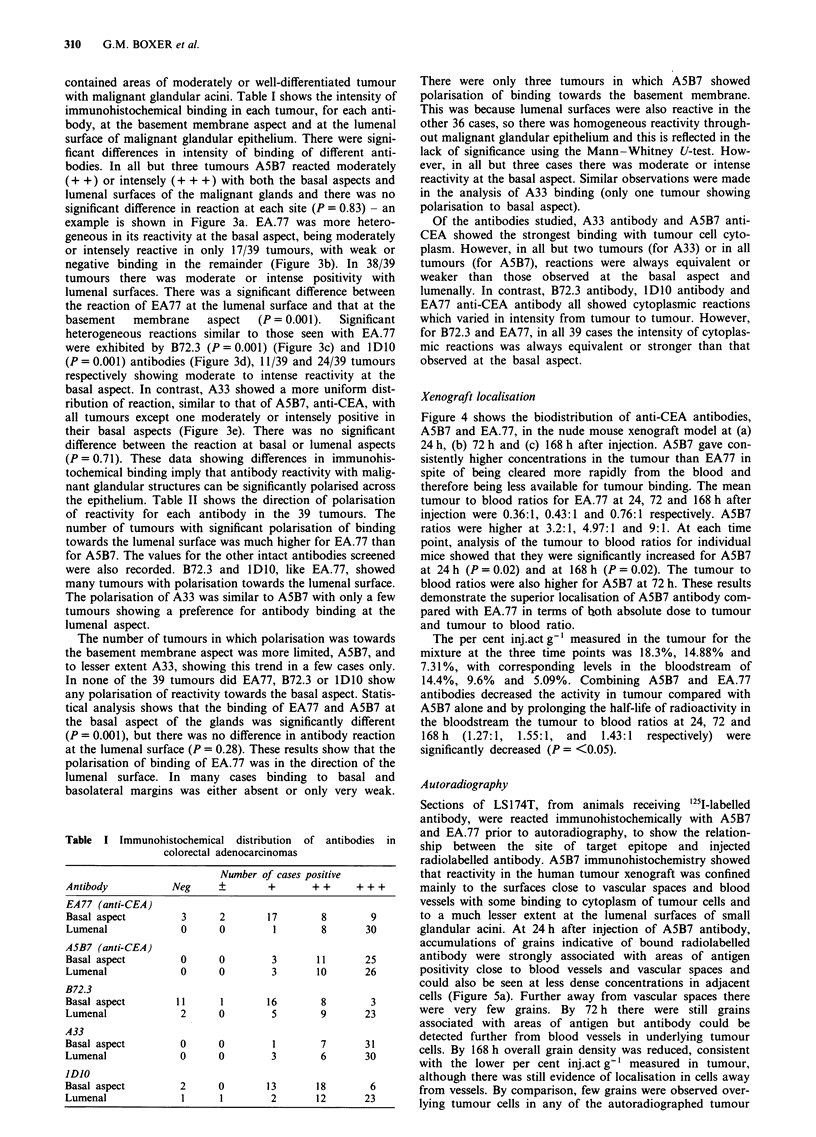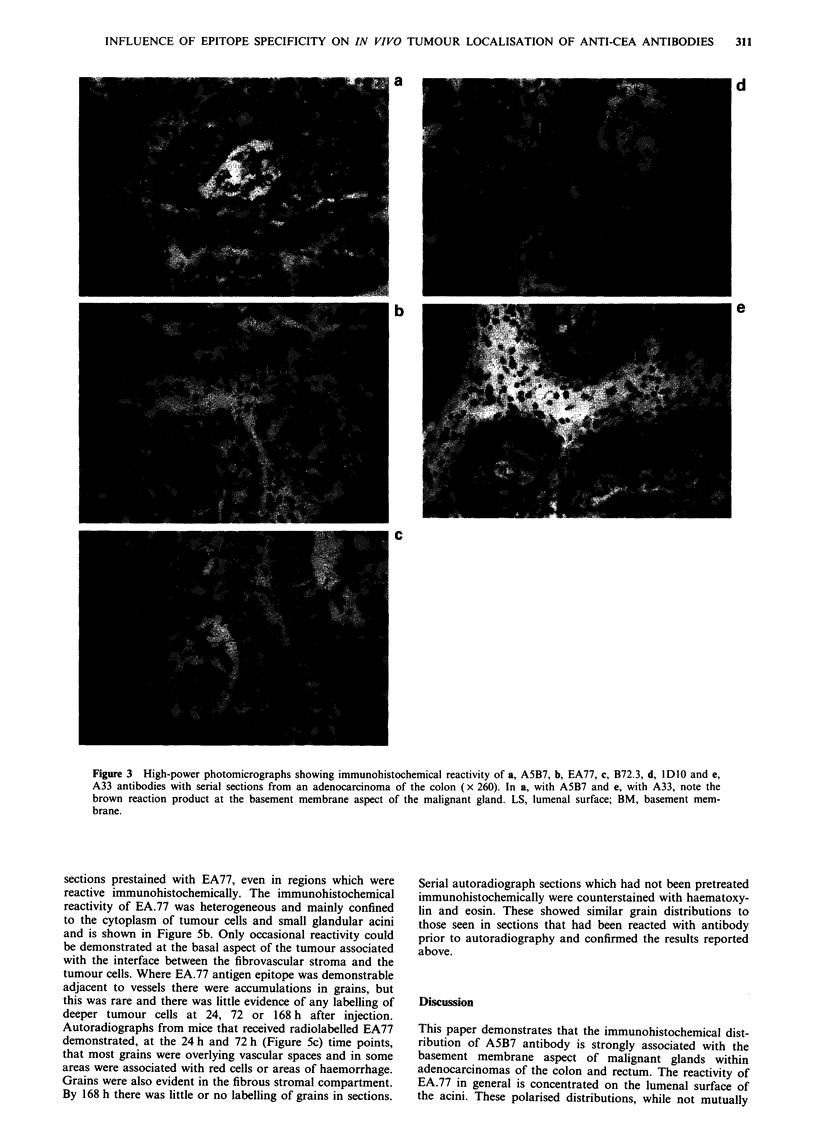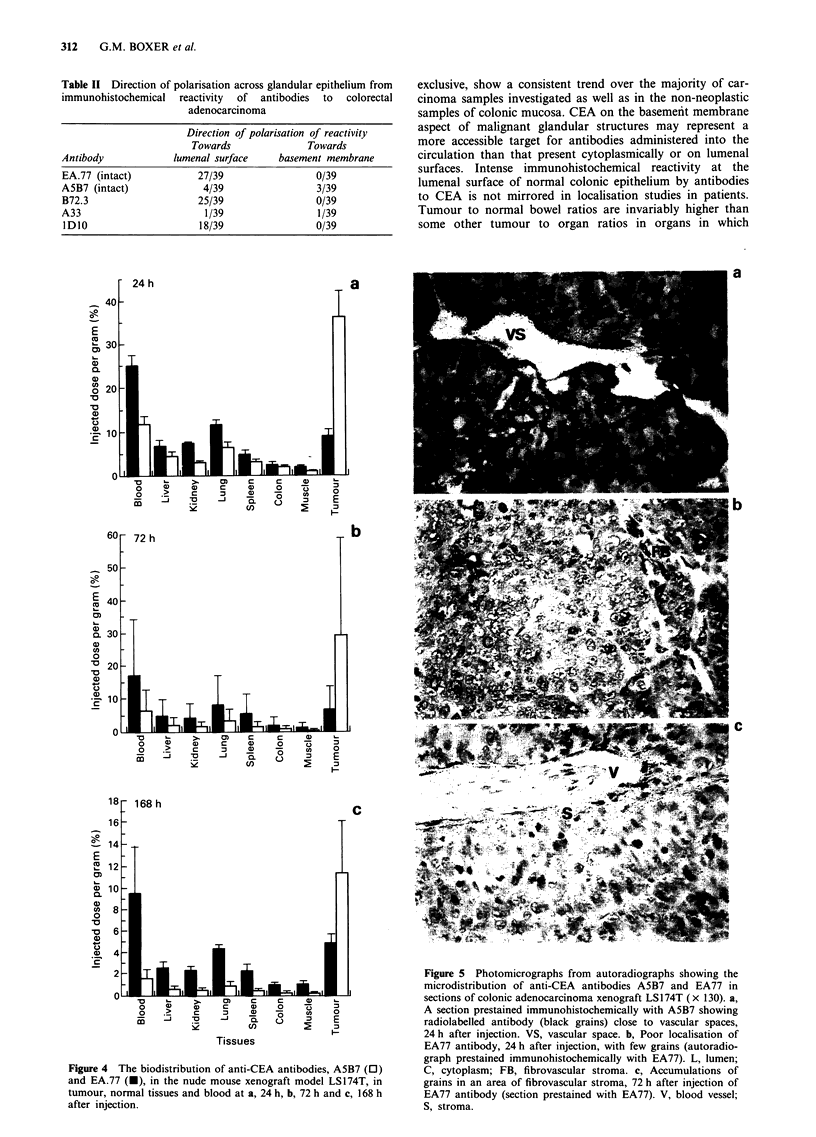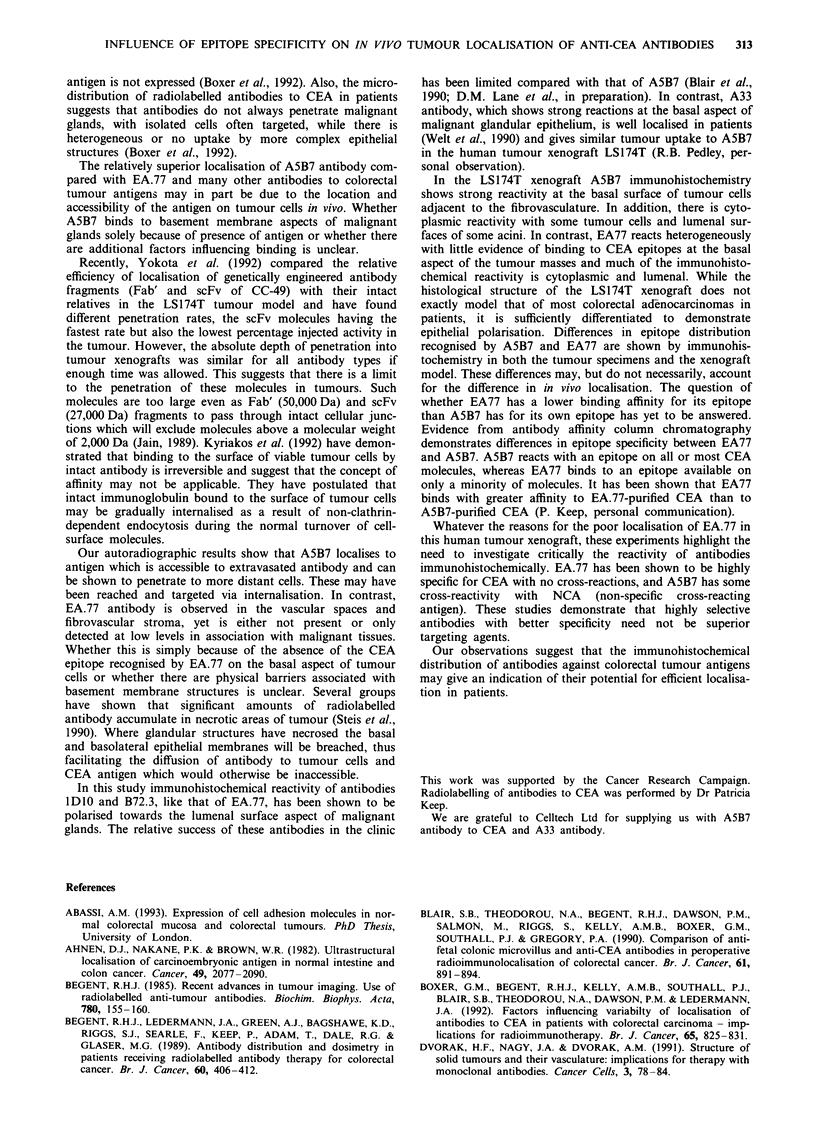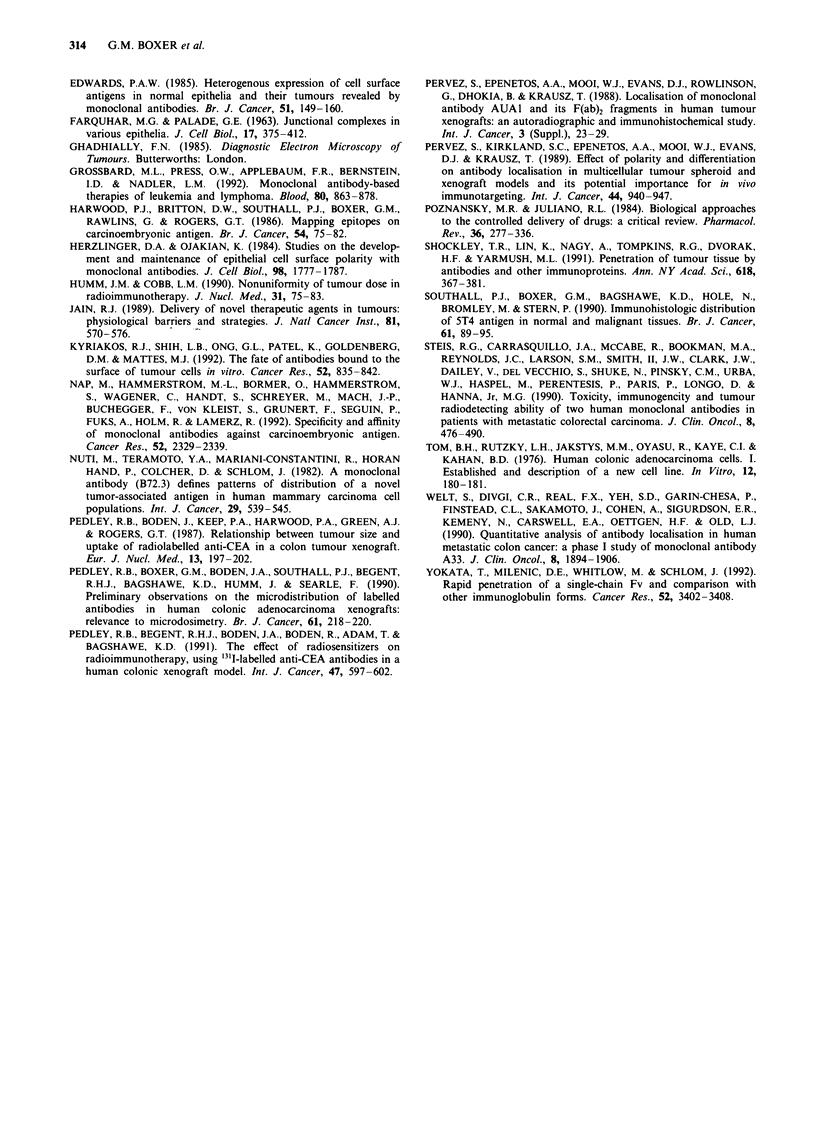# Localisation of monoclonal antibodies reacting with different epitopes on carcinoembryonic antigen (CEA)--implications for targeted therapy.

**DOI:** 10.1038/bjc.1994.56

**Published:** 1994-02

**Authors:** G. M. Boxer, A. M. Abassi, R. B. Pedley, R. H. Begent

**Affiliations:** University Department of Clinical Oncology, Royal Free Hospital School of Medicine, London, UK.

## Abstract

**Images:**


*Br. J. Cancer* (1994), **69**, 307-314

© Macmillan Press Ltd., 1994

### Localisation of monoclonal antibodies reacting with different epitopes on carcinoembryonic antigen (CEA) – implications for targeted therapy

G.M. Boxer, A.M. Abassi, R.B. Pedley & R.H.J. Begent

*Cancer Research Campaign Laboratories, University Department of Clinical Oncology, Royal Free Hospital School of Medicine, London NW3 2PF, UK.*

**Summary** Antibody targeting has potential for selective delivery of cancer therapy. However, there is a wide variation in the degree of antibody localisation in individual patients with colorectal adenocarcinoma. Colorectal adenocarcinomas are composed of glandular structures separated from fibrovascular stroma by a basal lamina which may represent a significant barrier to extravasated antibody. Basement membrane-associated CEA epitopes may be more accessible to antibodies than those which are cytoplasmic or lumenal. We have investigated, by immunohistochemistry and *in vivo* localisation, the extent to which distribution of antigen epitopes influences targeting. Two monoclonal antibodies (A5B7 and EA77) recognising non-overlapping CEA epitopes were reacted immunohistochemically with samples of 39 tumours. Intensity and site of reaction were assessed for basement membrane, cytoplasmic or lumenal surface association. $^{125}$I-labelled antibodies were injected into nude mice bearing LS174T tumour. Per cent injected activity per gram was measured in tumour and normal tissues, 24, 72 and 168 h later. Tissues reacted immunohistochemically for CEA were autoradiographed to assess the relationship of injected antibody to target antigen. Immunohistochemistry showed that A5B7 antibody favours basement membrane aspects of malignant glands; in contrast, EA77 concentrated generally on lumenal surfaces. *In vivo* localisation showed that per cent inj.act g$^{-1}$ in tumour for A5B7 reached 36.5% at 24 h. EA77 localised to a lesser extent (9.1% at 24 h), despite a longer circulatory half-life. Autoradiography combined with immunohistochemistry showed A5B7 reacting with antigen close to vasculature after 24 h, slowly penetrating deeper parts of the tumour by 72 h. In contrast, EA77 was confined mainly to fibrovascular stroma, showing little labelling of antigen-positive tumour cells. Localisation differences between A5B7 and EA77 may partly be due to accessibility of epitopes on tumour cells.

The administration of radiolabelled antibodies against tumour antigens is of value in the management of colorectal adenocarcinomas for both tumour localisation in diagnosis using external scintigraphy (Begent, 1985) and treatment using radioimmunoguided surgery (Blair *et al.*, 1990). The rationale of those techniques depends upon the selectivity of antibody for the target antigen expressed at the tumour site. However the success of antibody-targeted therapy depends not only upon the specificity of targeting but also on the ability to deliver tumoricidal amounts of therapy to the whole tumour (Humm & Cobb, 1990). Although partial and complete responses have been reported with antibodies directed against lymphomas (Grossbard *et al.*, 1992), reports of responses in colorectal cancer patients are limited (Begent *el al.*, 1989). This has been attributed, in part, to the heterogeneity of antigen distribution (Edwards, 1985) and is illustrated by the wide variation between patients in amount of antibody (per cent injected activity per kg) localising in tumour. Attempts to investigate which parameters are responsible for this variation have implicated a number of factors (Shockley *et al.*, 1991). There is some evidence that the cellular organisation in colonic adenocarcinomas may influence the efficiency with which antibody penetrates and is retained at the tumour site (Boxer *et al.*, 1992) and that a critically important factor is the inaccessibility of tumour antigen sites (Pervez *et al.*, 1988). Furthermore, Pervez *et al.* (1989) demonstrated that two antibodies directed against different antigens on colonic adenocarcinoma cells (one present lumenally and the other basolaterally associated), have different distributions *in vivo*. Colorectal adenocarcinomas are composed of complex glandular structures separated from fibrovascular stroma by a basal lamina. Although this basal lamina can be thin, interrupted or almost absent (Ghadhially, 1985), it may still represent a significant barrier to extravasated antibody molecules (Poznansky & Juliano, 1984; Dvorak *et al.*, 1991). Tight junctions separating apical and basolateral surfaces (Farquhar & Palade, 1963) are thought to play an important role in the maintenance of cell polarity (Herzlinger & Ojakian, 1984), and desmosomal intercellular junctions are present on lateral membranes. In malignant epithelium both these structures may hinder passage of antibody molecules.

Ahnen *et al.* (1982), demonstrated the localisation of CEA in normal intestine and colon cancer at the ultrastructural level using polyclonal antibodies. The results showed the association of CEA with basement membranes and basolateral surfaces of malignant colonic epithelium in tumours, in contrast to the apical distribution in normal colon, suggesting that the polarity of surface membrane components is disturbed in neoplasia. Antibodies which bind epitopes that are preferentially expressed on the lumenal surfaces of malignant acini or cytoplasmically may not be capable of reaching their target *in vivo*. Antibodies which bind to the basal and basolateral aspect of these glandular structures (Abassi, 1993) may have an advantage since the target is readily accessible to molecules diffusing through fibrovascular stroma after extravasation from the blood vessels.

This paper reports differences in the immunohistochemical distribution of two intact mouse monoclonal antibodies directed against non-overlapping epitopes on CEA. We have compared the relative efficiency of localisation (per cent injected activity in tumour and tumour to normal tissue ratios) of these antibodies, both as single agents and as a mixture, in the human tumour xenograft model LS174T. Their microdistribution has been studied autoradiographically.

### Materials and methods

### *Antibodies*

A5B7 and EA77 (anti-CEA antibodies) were obtained from the Cancer Research Campaign (CRC) Targeting Group, University Department of Clinical Oncology, Royal Free Hospital, London, UK. BA57 (Harwood *et al.*, 1986) and EA.77 (Nap *et al.*, 1992) are mouse monoclonal anti-CEA

Correspondence: G.M. Boxer.

Received 16 August 1993; and in revised form 29 September 1993.

IgG antibodies which react with non-overlapping epitopes on CEA. A5B7 (group 4) and EA.77 (group 2) have been characterised under the Gold classification, by Nap *et al.* (1992). Antibody 1D10 (obtained from the CRC Targeting Group) is directed against fetal microvillous membrane antigen and has been used clinically by Blair *et al.* (1990). B72.3 antibody to TAG-72 antigen (Nuti *et al.*, 1982) and an anti-colon carcinoma antigen antibody, A33 (Welt *et al.*, 1990), were obtained from Celltech. Control sections were processed without the primary antibody and with substitution of the primary antibody with mouse IgG.

*Immunofluorescence*

Studies were performed on samples of colonic adenocarcinoma and normal flanking tissue from ten patients (eight primary colonic adenocarcinomas and two metastatic adenocarcinomas). A case of squamous carcinoma from the anal margin was used as a control. Tissues were taken fresh from resection specimens and snap frozen in isopentane, cooled in liquid nitrogen. Cryostat sections (6 $\mu$m) were cut, air dried and then fixed in cold acetone for 5 min. Preliminary immunofluorescence studies were carried out using intact anti-CEA antibodies A5B7 and EA.77. Sections were reacted for 30 min with primary antibody (15 $\mu g\ ml^{-1}$) and washed in Tris-buffered saline (pH 7.4). They were then incubated for 30 min with FITC-labelled rabbit anti-mouse immunoglobulins (Vector Laboratories) diluted in 10% normal human serum, washed as before and then mounted in aqueous media (Vectashield). Sections were examined under fluorescence using the Zeiss Axiophot microscope and photographed.

*Immunohistochemistry*

A subsequent, more detailed characterisation of antibody binding was performed using an immunohistochemical study in a series of 39 additional samples of primary colorectal adenocarcinoma and five samples of non-neoplastic mucosa. Samples were snap frozen as before and cryostat sections prepared. An avidin–biotin–peroxidase technique was used as previously described (Southall *et al.*, 1990). Sections were incubated with primary antibody at a concentration of 15 $\mu g\ ml^{-1}$.

*Assessment of immunohistochemical reactivity*

Antibody reactivity was scored on an arbitrary scale of intensity ranging from + weak, + + moderate to + + + intense. Equivocal reactions were scored as ±. Immunohistochemical reactivity was assessed by recording the intensity of reaction on both the lumenal surface and basement membrane aspect of malignant glands. Intensity of reaction of the cytoplasm of the cells was noted (data not shown). For each antibody, at both the basal and lumenal aspects, the number of cases which showed + + + reactions were recorded. Similar data was generated for + +, +, ± and negative reactions.

For each antibody preparation, the number of tumours in which antibody binding showed a significant increase or gradient of reactivity from the basement membrane aspect to the lumenal surface was recorded. The number of cases where the direction of polarisation of binding was in the opposite direction – towards the basement membrane aspect – was also recorded.

*Statistical analysis*

Using the scoring system of + to + + +, values were attributed to the immunohistochemical reaction at the basement membrane aspect and at the lumenal surface of glandular structures, for each antibody. Negative or equivocal reactions scored 0, + scored 1, + + scored 2 and + + + scored 3. From these data we compared reactions at the basement membrane and lumenal surface using a Mann–Whitney *U*-test, in the 39 cases, to assess whether there was any significant difference for each antibody. Differences in the intensity of reaction between EA77 and A5B7, both at the basement membrane aspect and at the lumenal surface, were tested for significance using the same statistical test.

*Antibody localisation*

A human colon adenocarcinoma cell line LS174T (Tom *et al.*, 1976) was used to develop a xenograft model in female nude (*nu/nu*) mice by subcutaneous cell inoculation into the flank. Subsequent passaging was by continuous subcutaneous implantation of 1 $mm^3$ xenograft fragments. All mice used were 2–3 months old, and weighed between 20 and 25 g at the initiation of experiments. Nude mice (*nu/nu*) were implanted with human colonic adenocarcinoma xenograft LS174T (Pedley *et al.*, 1991) and used 3 weeks after passaging when the mean tumour volume was approximately 1 $cm^3$. Mice were injected i.v. with 10 $\mu$g of either $^{125}$I-labelled A5B7, EA.77 or a mixture of both. Antibodies were radiolabelled by the chloramine-T method over ice, to a specific activity of 1 $\mu Ci\ \mu g^{-1}$. After radioiodination anti-CEA antibodies, A5B7 and EA77, bound CEA antigen on a solid-phase radioimmunoassay. LS174T tumour is a moderately differentiated adenocarcinoma which grows as sheets of malignant cells within which numerous small acini are formed. In most tumours there are central areas of necrosis. The viable tumour is supported by fibrovascular stroma and there are some larger vascular spaces containing red cells. The connective tissue fibrovascular stroma is of mouse origin.

*Gamma counting of radioactivity*

Animals were sacrificed at 24, 72 and 168 h after injection (four animals per time point) and samples of tumour, blood, liver, lung, kidney, spleen, colon and muscle were taken. Samples were weighed, dissolved in 2 ml of 7 M potassium hydroxide and counted for gamma radioactivity in a gamma counter (Pharmacia – Wizard). Percentage injected activity per gram (per cent inj.act $g^{-1}$) of tissue was calculated as a mean of the values in four mice (Pedley *et al.*, 1987). Adjacent pieces of tissue were fixed in 10% formalin and processed for routine histology. Tumour to blood ratios were calculated.

Differences in tumour to blood ratio between EA77 and A5B7 antibodies, and between A5B7 and the mixture of antibodies (A5B7 + EA77), at each time point, were tested for statistical significance using the Mann–Whitney *U*-test.

*Autoradiography*

Five-micron sections of tumour and normal tissues were cut, mounted on glass slides pretreated with a 2% solution of 3, amino-triethoxysilane and air dried overnight at 37°C. After dewaxing in Inhibisol they were taken through graded alcohols to distilled water and covered with autoradiographic film. Briefly, in a darkroom slides were dipped for 8 s in a nuclear emulsion (K5, Ilford), diluted 1:1 in 2% glycerol (preheated to 42°C). They were air dried for 1 h and then placed in a darkbox with silica gel and left overnight. Slides were then transferred to lightproof darkboxes containing silica gel and exposed at 4°C for 4 weeks. Serial sections were first reacted immunohistochemically with A5B7 and EA.77 antibodies to CEA and then covered with autoradiographic emulsion. Autoradiographs were developed as previously described (Pedley *et al.*, 1990) and counterstained with haematoxylin and eosin. Immunohistochemically stained sections were counterstained with haematoxylin only.

### Results

*Immunofluorescence*

In normal colonic mucosa, both A5B7 and EA.77 antibodies were reactive with the apical region of epithelial cells in the upper third of the colonic crypt. Some CEA reactivity was observed at the lumenal surfaces of the middle and lower parts of the crypt, but the intensity of binding was weaker than that observed in the upper part of the crypt. Immunofluorescence binding of anti-CEA antibodies A5B7 and EA.77 in the ten primary colorectal adenocarcinomas showed three different distributions. These were (a) strong lumenal staining, (b) strong basal staining and (c) strong cytoplasmic reactions. The last was only observed in poorly differentiated tumours. The intensity of binding was variable. The binding of EA.77 was predominantly, and in some cases exclusively, confined to the lumenal surface of glandular acini, while A5B7 reacted primarily at the basement membrane aspect of glands. Figure 1a (EA.77) and b and c (A5B7) are representative photomicrographs illustrating the different distributions associated with the two anti-CEA antibodies in sections of colonic adenocarcinoma.

*Immunohistochemistry*

Immunohistochemical reactivity in sections of non-neoplastic colonic mucosa demonstrated differences in distribution of antibody binding. Antibody binding could be categorised into two groups. EA.77 (anti-CEA), B72.3 and 1D10 all showed strong reactions at the lumenal aspect of surface epithelium and with lumenal surfaces of goblet cells lining the crypts, cytoplasmic reactivity was weak. Figure 2a shows the reaction of anti-CEA antibody EA.77. A5B7 (anti-CEA) and A33 showed similar reactions but in addition bound strongly to basal and basolateral cell membranes throughout the crypt epithelium. Figure 2b (A5B7) and c (A33) show the additional reactions of antibody with basal aspects of the crypt epithelium. Cytoplasmic reactions were stronger, especially with A33, which also showed strong reactions with the basal surface epithelium. All of the 39 tumours studied

**Figure 1** Photomicrographs of immunofluorescence binding of anti-CEA antibodies in cyrostat sections of colonic adenocarcinoma: **a**, with EA77, showing lumenal surface reactivity of malignant glands (× 120) and **b** and **c** with A5B7 demonstrating **b**, basement membrane (× 120) and **c**, Lumenal surface reactivity (× 60). LS, lumenal surface; BM, basement membrane.

**Figure 2** Photomicrographs showing immunohistochemical reactivity of **a**, EA77, **b**, A5B7 and **c**, A33 antibodies with normal colonic mucosa (× 130).

contained areas of moderately or well-differentiated tumour with malignant glandular acini. Table I shows the intensity of immunohistochemical binding in each tumour, for each antibody, at the basement membrane aspect and at the lumenal surface of malignant glandular epithelium. There were significant differences in intensity of binding of different antibodies. In all but three tumours A5B7 reacted moderately (+ +) or intensely (+ + +) with both the basal aspects and lumenal surfaces of the malignant glands and there was no significant difference in reaction at each site ($P = 0.83$) – an example is shown in Figure 3a. EA.77 was more heterogeneous in its reactivity at the basal aspect, being moderately or intensely reactive in only 17/39 tumours, with weak or negative binding in the remainder (Figure 3b). In 38/39 tumours there was moderate or intense positivity with lumenal surfaces. There was a significant difference between the reaction of EA77 at the lumenal surface and that at the basement membrane aspect ($P = 0.001$). Significant heterogeneous reactions similar to those seen with EA.77 were exhibited by B72.3 ($P = 0.001$) (Figure 3c) and 1D10 ($P = 0.001$) antibodies (Figure 3d), 11/39 and 24/39 tumours respectively showing moderate to intense reactivity at the basal aspect. In contrast, A33 showed a more uniform distribution of reaction, similar to that of A5B7, anti-CEA, with all tumours except one moderately or intensely positive in their basal aspects (Figure 3e). There was no significant difference between the reaction at basal or lumenal aspects ($P = 0.71$). These data showing differences in immunohistochemical binding imply that antibody reactivity with malignant glandular structures can be significantly polarised across the epithelium. Table II shows the direction of polarisation of reactivity for each antibody in the 39 tumours. The number of tumours with significant polarisation of binding towards the lumenal surface was much higher for EA.77 than for A5B7. The values for the other intact antibodies screened were also recorded. B72.3 and 1D10, like EA.77, showed many tumours with polarisation towards the lumenal surface. The polarisation of A33 was similar to A5B7 with only a few tumours showing a preference for antibody binding at the lumenal aspect.

The number of tumours in which polarisation was towards the basement membrane aspect was more limited, A5B7, and to lesser extent A33, showing this trend in a few cases only. In none of the 39 tumours did EA77, B72.3 or 1D10 show any polarisation of reactivity towards the basal aspect. Statistical analysis shows that the binding of EA77 and A5B7 at the basal aspect of the glands was significantly different ($P = 0.001$), but there was no difference in antibody reaction at the lumenal surface ($P = 0.28$). These results show that the polarisation of binding of EA.77 was in the direction of the lumenal surface. In many cases binding to basal and basolateral margins was either absent or only very weak.

**Table I** Immunohistochemical distribution of antibodies in colorectal adenocarcinomas

| Antibody | Neg | *Number of cases positive* ± | + | + + | + + + |
|---|---|---|---|---|---|
| *EA77 (anti-CEA)* | | | | | |
| Basal aspect | 3 | 2 | 17 | 8 | 9 |
| Lumenal | 0 | 0 | 1 | 8 | 30 |
| *A5B7 (anti-CEA)* | | | | | |
| Basal aspect | 0 | 0 | 3 | 11 | 25 |
| Lumenal | 0 | 0 | 3 | 10 | 26 |
| *B72.3* | | | | | |
| Basal aspect | 11 | 1 | 16 | 8 | 3 |
| Lumenal | 2 | 0 | 5 | 9 | 23 |
| *A33* | | | | | |
| Basal aspect | 0 | 0 | 1 | 7 | 31 |
| Lumenal | 0 | 0 | 3 | 6 | 30 |
| *1D10* | | | | | |
| Basal aspect | 2 | 0 | 13 | 18 | 6 |
| Lumenal | 1 | 1 | 2 | 12 | 23 |

There were only three tumours in which A5B7 showed polarisation of binding towards the basement membrane. This was because lumenal surfaces were also reactive in the other 36 cases, so there was homogeneous reactivity throughout malignant glandular epithelium and this is reflected in the lack of significance using the Mann–Whitney *U*-test. However, in all but three cases there was moderate or intense reactivity at the basal aspect. Similar observations were made in the analysis of A33 binding (only one tumour showing polarisation to basal aspect).

Of the antibodies studied, A33 antibody and A5B7 anti-CEA showed the strongest binding with tumour cell cytoplasm. However, in all but two tumours (for A33) or in all tumours (for A5B7), reactions were always equivalent or weaker than those observed at the basal aspect and lumenally. In contrast, B72.3 antibody, 1D10 antibody and EA77 anti-CEA antibody all showed cytoplasmic reactions which varied in intensity from tumour to tumour. However, for B72.3 and EA77, in all 39 cases the intensity of cytoplasmic reactions was always equivalent or stronger than that observed at the basal aspect.

### *Xenograft localisation*

Figure 4 shows the biodistribution of anti-CEA antibodies, A5B7 and EA.77, in the nude mouse xenograft model at (a) 24 h, (b) 72 h and (c) 168 h after injection. A5B7 gave consistently higher concentrations in the tumour than EA77 in spite of being cleared more rapidly from the blood and therefore being less available for tumour binding. The mean tumour to blood ratios for EA.77 at 24, 72 and 168 h after injection were 0.36:1, 0.43:1 and 0.76:1 respectively. A5B7 ratios were higher at 3.2:1, 4.97:1 and 9:1. At each time point, analysis of the tumour to blood ratios for individual mice showed that they were significantly increased for A5B7 at 24 h ($P = 0.02$) and at 168 h ($P = 0.02$). The tumour to blood ratios were also higher for A5B7 at 72 h. These results demonstrate the superior localisation of A5B7 antibody compared with EA.77 in terms of both absolute dose to tumour and tumour to blood ratio.

The per cent inj.act $g^{-1}$ measured in the tumour for the mixture at the three time points was 18.3%, 14.88% and 7.31%, with corresponding levels in the bloodstream of 14.4%, 9.6% and 5.09%. Combining A5B7 and EA.77 antibodies decreased the activity in tumour compared with A5B7 alone and by prolonging the half-life of radioactivity in the bloodstream the tumour to blood ratios at 24, 72 and 168 h (1.27:1, 1.55:1, and 1.43:1 respectively) were significantly decreased ($P = <0.05$).

### *Autoradiography*

Sections of LS174T, from animals receiving $^{125}$I-labelled antibody, were reacted immunohistochemically with A5B7 and EA.77 prior to autoradiography, to show the relationship between the site of target epitope and injected radiolabelled antibody. A5B7 immunohistochemistry showed that reactivity in the human tumour xenograft was confined mainly to the surfaces close to vascular spaces and blood vessels with some binding to cytoplasm of tumour cells and to a much lesser extent at the lumenal surfaces of small glandular acini. At 24 h after injection of A5B7 antibody, accumulations of grains indicative of bound radiolabelled antibody were strongly associated with areas of antigen positivity close to blood vessels and vascular spaces and could also be seen at less dense concentrations in adjacent cells (Figure 5a). Further away from vascular spaces there were very few grains. By 72 h there were still grains associated with areas of antigen but antibody could be detected further from blood vessels in underlying tumour cells. By 168 h overall grain density was reduced, consistent with the lower per cent inj.act $g^{-1}$ measured in tumour, although there was still evidence of localisation in cells away from vessels. By comparison, few grains were observed overlying tumour cells in any of the autoradiographed tumour

**Figure 3** High-power photomicrographs showing immunohistochemical reactivity of **a**, A5B7, **b**, EA77, **c**, B72.3, **d**, 1D10 and **e**, A33 antibodies with serial sections from an adenocarcinoma of the colon (× 260). In **a**, with A5B7 and **e**, with A33, note the brown reaction product at the basement membrane aspect of the malignant gland. LS, lumenal surface; BM, basement membrane.

sections prestained with EA77, even in regions which were reactive immunohistochemically. The immunohistochemical reactivity of EA.77 was heterogeneous and mainly confined to the cytoplasm of tumour cells and small glandular acini and is shown in Figure 5b. Only occasional reactivity could be demonstrated at the basal aspect of the tumour associated with the interface between the fibrovascular stroma and the tumour cells. Where EA.77 antigen epitope was demonstrable adjacent to vessels there were accumulations in grains, but this was rare and there was little evidence of any labelling of deeper tumour cells at 24, 72 or 168 h after injection. Autoradiographs from mice that received radiolabelled EA77 demonstrated, at the 24 h and 72 h (Figure 5c) time points, that most grains were overlying vascular spaces and in some areas were associated with red cells or areas of haemorrhage. Grains were also evident in the fibrous stromal compartment. By 168 h there was little or no labelling of grains in sections.

Serial autoradiograph sections which had not been pretreated immunohistochemically were counterstained with haematoxylin and eosin. These showed similar grain distributions to those seen in sections that had been reacted with antibody prior to autoradiography and confirmed the results reported above.

### Discussion

This paper demonstrates that the immunohistochemical distribution of A5B7 antibody is strongly associated with the basement membrane aspect of malignant glands within adenocarcinomas of the colon and rectum. The reactivity of EA.77 in general is concentrated on the lumenal surface of the acini. These polarised distributions, while not mutually

**Table II** Direction of polarisation across glandular epithelium from immunohistochemical reactivity of antibodies to colorectal adenocarcinoma

| | *Direction of polarisation of reactivity* | |
|---|---|---|
| *Antibody* | *Towards lumenal surface* | *Towards basement membrane* |
| EA.77 (intact) | 27/39 | 0/39 |
| A5B7 (intact) | 4/39 | 3/39 |
| B72.3 | 25/39 | 0/39 |
| A33 | 1/39 | 1/39 |
| 1D10 | 18/39 | 0/39 |

exclusive, show a consistent trend over the majority of carcinoma samples investigated as well as in the non-neoplastic samples of colonic mucosa. CEA on the basement membrane aspect of malignant glandular structures may represent a more accessible target for antibodies administered into the circulation than that present cytoplasmically or on lumenal surfaces. Intense immunohistochemical reactivity at the lumenal surface of normal colonic epithelium by antibodies to CEA is not mirrored in localisation studies in patients. Tumour to normal bowel ratios are invariably higher than some other tumour to organ ratios in organs in which

**Figure 4** The biodistribution of anti-CEA antibodies, A5B7 (□) and EA.77 (■), in the nude mouse xenograft model LS174T, in tumour, normal tissues and blood at **a**, 24 h, **b**, 72 h and **c**, 168 h after injection.

**Figure 5** Photomicrographs from autoradiographs showing the microdistribution of anti-CEA antibodies A5B7 and EA77 in sections of colonic adenocarcinoma xenograft LS174T (× 130). **a**, A section prestained immunohistochemically with A5B7 showing radiolabelled antibody (black grains) close to vascular spaces, 24 h after injection. VS, vascular space. **b**, Poor localisation of EA77 antibody, 24 h after injection, with few grains (autoradiograph prestained immunohistochemically with EA77). L, lumen; C, cytoplasm; FB, fibrovascular stroma. **c**, Accumulations of grains in an area of fibrovascular stroma, 72 h after injection of EA77 antibody (section prestained with EA77). V, blood vessel; S, stroma.

antigen is not expressed (Boxer *et al.*, 1992). Also, the microdistribution of radiolabelled antibodies to CEA in patients suggests that antibodies do not always penetrate malignant glands, with isolated cells often targeted, while there is heterogeneous or no uptake by more complex epithelial structures (Boxer *et al.*, 1992).

The relatively superior localisation of A5B7 antibody compared with EA.77 and many other antibodies to colorectal tumour antigens may in part be due to the location and accessibility of the antigen on tumour cells *in vivo*. Whether A5B7 binds to basement membrane aspects of malignant glands solely because of presence of antigen or whether there are additional factors influencing binding is unclear.

Recently, Yokota *et al.* (1992) compared the relative efficiency of localisation of genetically engineered antibody fragments (Fab' and scFv of CC-49) with their intact relatives in the LS174T tumour model and have found different penetration rates, the scFv molecules having the fastest rate but also the lowest percentage injected activity in the tumour. However, the absolute depth of penetration into tumour xenografts was similar for all antibody types if enough time was allowed. This suggests that there is a limit to the penetration of these molecules in tumours. Such molecules are too large even as Fab' (50,000 Da) and scFv (27,000 Da) fragments to pass through intact cellular junctions which will exclude molecules above a molecular weight of 2,000 Da (Jain, 1989). Kyriakos *et al.* (1992) have demonstrated that binding to the surface of viable tumour cells by intact antibody is irreversible and suggest that the concept of affinity may not be applicable. They have postulated that intact immunoglobulin bound to the surface of tumour cells may be gradually internalised as a result of non-clathrin-dependent endocytosis during the normal turnover of cell-surface molecules.

Our autoradiographic results show that A5B7 localises to antigen which is accessible to extravasated antibody and can be shown to penetrate to more distant cells. These may have been reached and targeted via internalisation. In contrast, EA.77 antibody is observed in the vascular spaces and fibrovascular stroma, yet is either not present or only detected at low levels in association with malignant tissues. Whether this is simply because of the absence of the CEA epitope recognised by EA.77 on the basal aspect of tumour cells or whether there are physical barriers associated with basement membrane structures is unclear. Several groups have shown that significant amounts of radiolabelled antibody accumulate in necrotic areas of tumour (Steis *et al.*, 1990). Where glandular structures have necrosed the basal and basolateral epithelial membranes will be breached, thus facilitating the diffusion of antibody to tumour cells and CEA antigen which would otherwise be inaccessible.

In this study immunohistochemical reactivity of antibodies 1D10 and B72.3, like that of EA.77, has been shown to be polarised towards the lumenal surface aspect of malignant glands. The relative success of these antibodies in the clinic has been limited compared with that of A5B7 (Blair *et al.*, 1990; D.M. Lane *et al.*, in preparation). In contrast, A33 antibody, which shows strong reactions at the basal aspect of malignant glandular epithelium, is well localised in patients (Welt *et al.*, 1990) and gives similar tumour uptake to A5B7 in the human tumour xenograft LS174T (R.B. Pedley, personal observation).

In the LS174T xenograft A5B7 immunohistochemistry shows strong reactivity at the basal surface of tumour cells adjacent to the fibrovasculature. In addition, there is cytoplasmic reactivity with some tumour cells and lumenal surfaces of some acini. In contrast, EA77 reacts heterogeneously with little evidence of binding to CEA epitopes at the basal aspect of the tumour masses and much of the immunohistochemical reactivity is cytoplasmic and lumenal. While the histological structure of the LS174T xenograft does not exactly model that of most colorectal adenocarcinomas in patients, it is sufficiently differentiated to demonstrate epithelial polarisation. Differences in epitope distribution recognised by A5B7 and EA77 are shown by immunohistochemistry in both the tumour specimens and the xenograft model. These differences may, but do not necessarily, account for the difference in *in vivo* localisation. The question of whether EA77 has a lower binding affinity for its epitope than A5B7 has for its own epitope has yet to be answered. Evidence from antibody affinity column chromatography demonstrates differences in epitope specificity between EA77 and A5B7. A5B7 reacts with an epitope on all or most CEA molecules, whereas EA77 binds to an epitope available on only a minority of molecules. It has been shown that EA77 binds with greater affinity to EA.77-purified CEA than to A5B7-purified CEA (P. Keep, personal communication).

Whatever the reasons for the poor localisation of EA.77 in this human tumour xenograft, these experiments highlight the need to investigate critically the reactivity of antibodies immunohistochemically. EA.77 has been shown to be highly specific for CEA with no cross-reactions, and A5B7 has some cross-reactivity with NCA (non-specific cross-reacting antigen). These studies demonstrate that highly selective antibodies with better specificity need not be superior targeting agents.

Our observations suggest that the immunohistochemical distribution of antibodies against colorectal tumour antigens may give an indication of their potential for efficient localisation in patients.

This work was supported by the Cancer Research Campaign. Radiolabelling of antibodies to CEA was performed by Dr Patricia Keep.

We are grateful to Celltech Ltd for supplying us with A5B7 antibody to CEA and A33 antibody.

### References

ABASSI, A.M. (1993). Expression of cell adhesion molecules in normal colorectal mucosa and colorectal tumours. *PhD Thesis*, University of London.

AHNEN, D.J., NAKANE, P.K. & BROWN, W.R. (1982). Ultrastructural localisation of carcinoembryonic antigen in normal intestine and colon cancer. *Cancer*, **49**, 2077–2090.

BEGENT, R.H.J. (1985). Recent advances in tumour imaging. Use of radiolabelled anti-tumour antibodies. *Biochim. Biophys. Acta*, **780**, 155–160.

BEGENT, R.H.J., LEDERMANN, J.A., GREEN, A.J., BAGSHAWE, K.D., RIGGS, S.J., SEARLE, F., KEEP, P., ADAM, T., DALE, R.G. & GLASER, M.G. (1989). Antibody distribution and dosimetry in patients receiving radiolabelled antibody therapy for colorectal cancer. *Br. J. Cancer*, **60**, 406–412.

BLAIR, S.B., THEODOROU, N.A., BEGENT, R.H.J., DAWSON, P.M., SALMON, M., RIGGS, S., KELLY, A.M.B., BOXER, G.M., SOUTHALL, P.J. & GREGORY, P.A. (1990). Comparison of anti-fetal colonic microvillus and anti-CEA antibodies in peroperative radioimmunolocalisation of colorectal cancer. *Br. J. Cancer*, **61**, 891–894.

BOXER, G.M., BEGENT, R.H.J., KELLY, A.M.B., SOUTHALL, P.J., BLAIR, S.B., THEODOROU, N.A., DAWSON, P.M. & LEDERMANN, J.A. (1992). Factors influencing variabilty of localisation of antibodies to CEA in patients with colorectal carcinoma – implications for radioimmunotherapy. *Br. J. Cancer*, **65**, 825–831.

DVORAK, H.F., NAGY, J.A. & DVORAK, A.M. (1991). Structure of solid tumours and their vasculature: implications for therapy with monoclonal antibodies. *Cancer Cells*, **3**, 78–84.

EDWARDS, P.A.W. (1985). Heterogenous expression of cell surface antigens in normal epithelia and their tumours revealed by monoclonal antibodies. *Br. J. Cancer*, **51**, 149-160.

FARQUHAR, M.G. & PALADE, G.E. (1963). Junctional complexes in various epithelia. *J. Cell Biol.*, **17**, 375-412.

GHADHIALLY, F.N. (1985). *Diagnostic Electron Microscopy of Tumours*. Butterworths: London.

GROSSBARD, M.L., PRESS, O.W., APPLEBAUM, F.R., BERNSTEIN, I.D. & NADLER, L.M. (1992). Monoclonal antibody-based therapies of leukemia and lymphoma. *Blood*, **80**, 863-878.

HARWOOD, P.J., BRITTON, D.W., SOUTHALL, P.J., BOXER, G.M., RAWLINS, G. & ROGERS, G.T. (1986). Mapping epitopes on carcinoembryonic antigen. *Br. J. Cancer*, **54**, 75-82.

HERZLINGER, D.A. & OJAKIAN, K. (1984). Studies on the development and maintenance of epithelial cell surface polarity with monoclonal antibodies. *J. Cell Biol.*, **98**, 1777-1787.

HUMM, J.M. & COBB, L.M. (1990). Nonuniformity of tumour dose in radioimmunotherapy. *J. Nucl. Med.*, **31**, 75-83.

JAIN, R.J. (1989). Delivery of novel therapeutic agents in tumours: physiological barriers and strategies. *J. Natl Cancer Inst.*, **81**, 570-576.

KYRIAKOS, R.J., SHIH, L.B., ONG, G.L., PATEL, K., GOLDENBERG, D.M. & MATTES, M.J. (1992). The fate of antibodies bound to the surface of tumour cells *in vitro*. *Cancer Res.*, **52**, 835-842.

NAP, M., HAMMERSTROM, M.-L., BORMER, O., HAMMERSTROM, S., WAGENER, C., HANDT, S., SCHREYER, M., MACH, J.-P., BUCHEGGER, F., VON KLEIST, S., GRUNERT, F., SEGUIN, P., FUKS, A., HOLM, R. & LAMERZ, R. (1992). Specificity and affinity of monoclonal antibodies against carcinoembryonic antigen. *Cancer Res.*, **52**, 2329-2339.

NUTI, M., TERAMOTO, Y.A., MARIANI-CONSTANTINI, R., HORAN HAND, P., COLCHER, D. & SCHLOM, J. (1982). A monoclonal antibody (B72.3) defines patterns of distribution of a novel tumor-associated antigen in human mammary carcinoma cell populations. *Int. J. Cancer*, **29**, 539-545.

PEDLEY, R.B., BODEN, J., KEEP, P.A., HARWOOD, P.A., GREEN, A.J. & ROGERS, G.T. (1987). Relationship between tumour size and uptake of radiolabelled anti-CEA in a colon tumour xenograft. *Eur. J. Nucl. Med.*, **13**, 197-202.

PEDLEY, R.B., BOXER, G.M., BODEN, J.A., SOUTHALL, P.J., BEGENT, R.H.J., BAGSHAWE, K.D., HUMM, J. & SEARLE, F. (1990). Preliminary observations on the microdistribution of labelled antibodies in human colonic adenocarcinoma xenografts: relevance to microdosimetry. *Br. J. Cancer*, **61**, 218-220.

PEDLEY, R.B., BEGENT, R.H.J., BODEN, J.A., BODEN, R., ADAM, T. & BAGSHAWE, K.D. (1991). The effect of radiosensitizers on radioimmunotherapy, using $^{131}$I-labelled anti-CEA antibodies in a human colonic xenograft model. *Int. J. Cancer*, **47**, 597-602.

PERVEZ, S., EPENETOS, A.A., MOOI, W.J., EVANS, D.J., ROWLINSON, G., DHOKIA, B. & KRAUSZ, T. (1988). Localisation of monoclonal antibody AUA1 and its F(ab)$_2$ fragments in human tumour xenografts: an autoradiographic and immunohistochemical study. *Int. J. Cancer*, **3** (Suppl.), 23-29.

PERVEZ, S., KIRKLAND, S.C., EPENETOS, A.A., MOOI, W.J., EVANS, D.J. & KRAUSZ, T. (1989). Effect of polarity and differentiation on antibody localisation in multicellular tumour spheroid and xenograft models and its potential importance for *in vivo* immunotargeting. *Int. J. Cancer*, **44**, 940-947.

POZNANSKY, M.R. & JULIANO, R.L. (1984). Biological approaches to the controlled delivery of drugs: a critical review. *Pharmacol. Rev.*, **36**, 277-336.

SHOCKLEY, T.R., LIN, K., NAGY, A., TOMPKINS, R.G., DVORAK, H.F. & YARMUSH, M.L. (1991). Penetration of tumour tissue by antibodies and other immunoproteins. *Ann. NY Acad. Sci.*, **618**, 367-381.

SOUTHALL, P.J., BOXER, G.M., BAGSHAWE, K.D., HOLE, N., BROMLEY, M. & STERN, P. (1990). Immunohistologic distribution of 5T4 antigen in normal and malignant tissues. *Br. J. Cancer*, **61**, 89-95.

STEIS, R.G., CARRASQUILLO, J.A., MCCABE, R., BOOKMAN, M.A., REYNOLDS, J.C., LARSON, S.M., SMITH, II, J.W., CLARK, J.W., DAILEY, V., DEL VECCHIO, S., SHUKE, N., PINSKY, C.M., URBA, W.J., HASPEL, M., PERENTESIS, P., PARIS, P., LONGO, D. & HANNA, Jr, M.G. (1990). Toxicity, immunogencity and tumour radiodetecting ability of two human monoclonal antibodies in patients with metastatic colorectal carcinoma. *J. Clin. Oncol.*, **8**, 476-490.

TOM, B.H., RUTZKY, L.H., JAKSTYS, M.M., OYASU, R., KAYE, C.I. & KAHAN, B.D. (1976). Human colonic adenocarcinoma cells. I. Established and description of a new cell line. *In Vitro*, **12**, 180-181.

WELT, S., DIVGI, C.R., REAL, F.X., YEH, S.D., GARIN-CHESA, P., FINSTEAD, C.L., SAKAMOTO, J., COHEN, A., SIGURDSON, E.R., KEMENY, N., CARSWELL, E.A., OETTGEN, H.F. & OLD, L.J. (1990). Quantitative analysis of antibody localisation in human metastatic colon cancer: a phase I study of monoclonal antibody A33. *J. Clin. Oncol.*, **8**, 1894-1906.

YOKATA, T., MILENIC, D.E., WHITLOW, M. & SCHLOM, J. (1992). Rapid penetration of a single-chain Fv and comparison with other immunoglobulin forms. *Cancer Res.*, **52**, 3402-3408.